# Study protocol: a mixed methods study to assess mental health recovery, shared decision-making and quality of life (Plan4Recovery)

**DOI:** 10.1186/s12913-016-1640-y

**Published:** 2016-08-17

**Authors:** Michael Coffey, Ben Hannigan, Alan Meudell, Julian Hunt, Deb Fitzsimmons

**Affiliations:** 1 Department of Public Health, Policy and Social Sciences, Swansea University, Swansea, SA2 8PP UK; 2 School of Healthcare Sciences, Cardiff University, Cardiff, UK; 3 Swansea Centre for Health Economics, Swansea University, Swansea, UK; 4 Caerphilly Mind, Caerphilly, UK

**Keywords:** Recovery, Shared decision making, Social support, Mental health, Quality of life, Wales

## Abstract

**Background:**

Recovery in mental health care is complex, highly individual and can be facilitated by a range of professional and non-professional support. In this study we will examine how recovery from mental health problems is promoted in non-medical settings. We hypothesise a relationship between involvement in decisions about care, social support and recovery and quality of life outcomes.

**Methods:**

We will use standardised validated instruments of involvement in decision-making, social contacts, recovery and quality of life with a random sample of people accessing non-statutory mental health social care services in Wales. We will add to this important information with detailed one to one case study interviews with people, their family members and their support workers. We will use a series of these interviews to examine how people build recovery over time to help us understand more about their involvement in decisions and the social links they build.

**Discussion:**

We want to see how being involved in decisions about care and the social links people have are related to recovery and quality of life for people with experience of using mental health support services. We want to understand the different perspectives of the people involved in making recovery possible. We will use this information to guide further studies of particular types of social interventions and their use in helping recovery from mental health problems.

## Background

Mental illness is common (about 1 in 4 people affected), occurs across the lifespan and impacts on all areas of everyday life. Conditions are often chronic and significantly affect relationships, employment, economic activity, housing, education and reduce contributions to wider social capital. The cost of mental illness in monetary terms is considerable, estimated at £7.2 billion in 2007/08 in Wales [1]. Better co-ordination and planning of care may improve outcomes, help to build recovery and enable greater participation in daily life.

Recovery is defined as “regaining mental health to the maximum extent possible and achieving the best possible quality of life, lived as independently as possible” ([2] p.8). This working definition is highly consistent with Anthony’s universally accepted description of recovery as “a way of living a satisfying, hopeful and contributing life even within the limitations caused by illness” ([3] p.15).

Collaborative or shared decision-making appears to aid recovery and requires that both parties exchange information, make attempts to reach consensus in treatment goals and agree on treatment plans [4]. The benefits associated with shared decision-making include reduced patient distress, improved functional status, improved satisfaction with services and a greater sense of control [5].

### Mental health and social support/Social connectedness

The notion of social support includes structural characteristics of an individual’s social networks as well as functional aspects of social relationships among group members. Structural conditions may be understood as the social context in which social interactions take place and the interchange of resources, information, goods or services are understood as the functional aspect of a given network. Social support can be positioned within a broad semantic network that covers social integration, social networks, social relationships and other concepts referring both to the individual’s social behaviour (both overt and covert) and their interaction with a group, community or society [6].

Social support can be understood as a form of social capital [7] to draw upon to assist coping with the stresses of daily life. The concept of social capital offers a possible explanatory and functional model for addressing the range of challenges faced by people with mental ill health living within communities. Support derived from networks incorporates material and emotional support and consists of both formal and informal social ties [8, 9]. Social ties can help people to adapt, make adjustments and improve recovery times in many conditions [10]. There remains limited research regarding the ways in which social support from multiple sources (family, peers and community) influence processes of recovery from mental ill health. Such an understanding is crucial so that researchers and mental health professionals can make informed decisions regarding where to focus care and treatment interventions [11]. Moreover, little attention has been afforded to the ways in which social support relates to the positive indicators of mental health recovery, such as hope for the future. To address these gaps, our study seeks to examine the relationship between social support from multiple sources and its role in enabling people’s recovery.

### Sharing in decisions about care

It has been suggested that as health care moves into the 21st century the focus of much medical intervention has moved away from the treatment of acute illness and into the realm of palliative treatments for chronic or longer term conditions [12]. Treatment of chronic conditions often involves maintaining engagement between people using services and health and social care workers with a clear imperative to achieve consensus and agreement about treatment goals as a means to ensure care options are congruent with the values and lifestyle of the individual. Shared decision making (SDM) has been seen as one means to this end. SDM is an interactional process involving at least two people (usually the person and a health or social care support worker) where there is sharing of information about treatment and care evidence alongside consideration of attitudes to risk. Steps are then taken by both parties to reach a consensus and agreement about the preferred treatment decision [4]. SDM lies along a continuum of forms of decision making in health and social care settings which range from paternalistic to informed choice approaches [13]. Emerging evidence across the medical spectrum points to the utility of the concept in a range of conditions in achieving greater patient involvement, improved satisfaction with treatment and better health outcomes [14].

In the context of mental health care shared decision making remains relatively new, untried and may present significant challenges for people using services and those providing them. There are three conditions needed for the implementation of SDM in health and social care contexts as follows,

1. Access to evidence based information on treatment options,
2. Guidance on how to weigh up the pros and cons of different options
3. A supportive culture that facilitates patient engagement [15].

The evidence for the presence of these conditions needs to be interrogated in relation to mental health care. It is questionable for instance how we should take the notion of sufficient evidence for treatment options in mental health care especially when the most frequently available treatments are pharmacological only and have doubtful efficacy or considerable dangers [16]. Decision making in relation to medication in mental health care is complex and casts SDM as an alternative to traditional paternalist views of medication compliance in these settings [17]. Mental health settings seeking to promote recovery via patient centred care that promotes involvement and engagement seem to struggle to achieve recovery oriented practice [18]. While some services seek to present service models that provide these conditions it appears that mismatches between staff and patient understandings of the concept of recovery may work against these intentions [19]. How people are further involved by other agencies on decisions related to non-treatment but condition-relevant decisions remains largely unknown. The evidence to date is that collaborative and shared involvement in the process of care is patchy with many users unsure of who their care co-ordinator is and uncertain about the content of care plans [20–23]. We would like to examine the extent of collaborative involvement in decisions about care from the perspective of service users and how this is associated with patient reported recovery and quality of life outcomes.

### Project description

This study will investigate the role of social approaches in promoting recovery from mental health problems. For the purposes of this research we are interested in examining involvement in decisions about care and the extent of networks built by the person within their social settings to understand how these relate to recovery and quality of life outcomes.

Our research question is: In what ways, if any, do social interventions promote recovery from mental health problems?

We hypothesise that care provided with an emphasis on social interventions will be associated with improved quality of life and recovery outcomes for people with mental health problems. We further hypothesise that there will be a positive association between recovery, involvement, size and depth of social networks and quality of life.

Due to the disparate nature of current service provision and the inchoate nature of the evidence for involvement in decisions and its relationship with recovery in mental health care, an observational study that undertakes exploratory analysis of likely variables is best suited to develop the empirical case for designing future controlled studies. In this study we wish to examine the use of these specific social interventions to better understand their relevance to outcomes and to provide the scientific underpinning for further studies.

#### Aim and objectives

The aim of this study is to provide new evidence on social approaches to recovery in mental health problems from the perspective of those using services.

This study will therefore examine whether shared decision making and social network ties are related to better outcomes and contribute to recovery and quality of life.

#### Our objectives are

1. To investigate involvement in decisions in planning of mental health care for social care users and their relationship to recovery and quality of life outcomes.
2. To examine the support networks of social care users and their relationship to patient reported recovery and quality of life outcomes.
3. To assess the relationship between recovery, involvement in decisions, social networks and quality of life.
4. To use detailed case studies to examine how social interventions are experienced by those receiving and providing them.

## Design and method

The study will adopt a mixed-methods design incorporating quantitative data collected at two points using reliable, validated, standardised measures of involvement in decisions, extent of social network links, recovery and quality of life alongside qualitative data collected in case studies. The standardised measures will help us to see the extent of involvement, how recovery is supported over time and allows us to measure outcomes. These data will be supplemented by case studies to provide a more in-depth understanding of process, experiences and outcomes, from the perspectives of individuals, those working with them and their significant others.

### Recruitment and sampling

There are multiple providers of mental health and social care services across public and voluntary sectors in Wales. It is our intention to access a sample of people through a mental health charity provider currently providing services to approximately 1400 people across Wales. The organisation’s client database will be anonymised to provide a sampling frame from which a random sample will be drawn.

Data will be collected in two overlapping stages. All potential participants will be given written information on the study and their informed consent sought. Our inclusion criteria will include adults of working age, who have been in receipt of mental health care, will include new (<2 years) and established cases (>2 years) and focus on those who have capacity to consent. Exclusion criteria will include people identified by support staff as being in current crisis or psychotic episode and those who are currently receiving care in hospital.

First, we will access a random sample (*n* = 200, + 20 % allowance for attrition) of people using social care services. We have calculated the size of this sample to ensure it is sufficiently powered to allow us to control for a range of variables [24]. In addition we have made allowances for possible sample attrition and non-response across the life of the study. This sample will be asked to consent to participate in completing standardised measures of recovery, involvement in decisions, social network support and quality of life. This will allow us to assess the relationship between social approaches and outcomes for the individual. Each participant will receive an invitation to participate along with a package of reliable and valid standardised measures as set out below. We will ask participants to do this twice, once at intake and once again 6–8 months into the study.

Second, we will through our partners in collaborating agencies seek to purposively sample (*n* = 12-15) individuals to follow their care trajectories for a period of not more than 6 months with the aim of building as complete a picture as possible of their individual recoveries. This will involve qualitative one to one interviews with the person, their support worker if they have one, family members or significant others. In our interviews we will ask about experiences of involvement, social networks and recovery. We will establish a lived experience advisory panel for the purposes of providing advice on the precise wording and extent of these questions so that our work is informed by these perspectives. We will also, with permission, observe care planning meetings, examine care and treatment plans and other relevant documentation related to their planned recovery. We will aim to recruit both men and women, with new or recent contact with services and accessing primary, secondary or non-statutory services. Our aim is to select a sample representing the range of people engaged in recovery from mental health problems.

### Survey

For the first stage our questionnaire pack will include:

- An information sheet on the study
- Lubben Social Network Scale (LSNS) [10] for charting social networks of participants. This is an 18-item validated scale measuring size, closeness and frequency of contact with friends, family and neighbours. Social networks will be assessed twice, once at intake and again at 6–8 months using the LSNS.
- The Process of Recovery Questionnaire (QPR) [25] is a 22-item scale which measures both intrapersonal and interpersonal tasks involved in recovery. It is strongly associated with general psychological wellbeing, quality of life and empowerment which are all linked with recovery from mental illness. Recovery has been found to be inversely related to symptoms and positively correlated with quality of life and empowerment [26]. The QPR will enable us to measure recovery outcomes associated with interventions over time. Recovery will be assessed twice, once at intake and again at 6–8 months using the QPR.
- Decision Conflict Scale [27] is a 16-item measure recommended by the shared decision making programme [28] and a recent Cochrane review [14] as the most widely used empirically derived and validated measure to assess decision process and outcome. The scale elicits information regarding the decision maker’s uncertainty in making a choice, modifiable factors contributing to uncertainty (lack of information, unclear values and inadequate social support) and perceived effective decision making. Decision making will be assessed twice, once at intake and again at 6–8 months.
- Manchester Short Assessment of Quality of Life Scale (MANSA) to measure quality of life [29]. This is a brief operational measure of quality of life across 8 domains. It includes objective and subjective components to measure the person’s view on life, work, education, leisure, safety, health, finance, family, social and living situation. Quality of life will be assessed twice, once at intake and again at 6–8 months using the MANSA.

For each completed questionnaire pack we will, with the help of service colleagues, complete the following:

- To enable us to understand the case-mix in different settings we will use the Matching Resources to Care (MARC2) measure. MARC2 is a multi-dimensional reliable and validated measure of severity and case complexity of mental health problems [30]. MARC2 incorporates components related to social situation, illness severity, risk and social exclusion [31]. This measure includes a brief demographic questionnaire to allow us to describe our sample and includes information on age, gender, length of contact with services, presenting mental health problem, living situation, employment situation and accommodation status. All participants recruited to the study will be assessed on the MARC2 once at intake to the study.

### Case studies

We will purposively select individuals and invite them to allow us to follow their unfolding care trajectories [32, 33] over a period of 4–6 months. We will examine everyday realities of using and providing social interventions for recovery through in-depth ethnographic case studies of each individual’s journey through the system of care. We will be particularly interested in hearing about involvement in decision making and increasing social networks to support recovery. To do this we will seek permission to observe care planning meetings where they occur, carry out document analysis of care plans and recovery policies and conduct research interviews with key stakeholders including the person, their carers, their workers and other key individuals. We envisage generating a significant amount of data from approximately 40 research interviews plus observations and document analysis. Informed by a theory of street level bureaucracy [34] we will examine what happens for individuals building recoveries when they encounter and are served by autonomous, discretionary, workers or services at the micro-level in the context of wider macro level system change. Our aim is to build as complete a picture as possible based upon multiple perspectives approach [35] to enable use to examine differing accounts of recovery.

### Ethical issues

Participants in our study were in many cases past or present users of health and social care organisations. We therefore applied for research ethics permissions via the NHS Research Ethics Service and received ethics clearance from West of Scotland Research Ethics Service on 18th March 2014 (REC ref: 14/WS/0063). We additionally applied for permissions through the NISCHR Permissions Co-ordinating Unit for research access to National Health Service organisations across Wales where necessary. NHS Research and Development applications for Health Boards were then completed for the purposes of gaining permission to NHS case notes to complete one standardised measure of complexity.

The main recruitment source for our study was via a national mental health charity database. Permission to use an anonymised database was secured via the chief executive of the organisation. Our initial focus is to ensure that participants selected for this study have the capacity to offer informed consent. This can be a concern with vulnerable groups and in those with mental health problems where capacity can be temporarily impaired. Where necessary we will consult directly with agency workers on this matter though for the most part we will treat those who chose to return a completed survey pack to us as having capacity to offer informed consent for that part of the study.

All participants will be given detailed written information on the study and for those participating in the case study interviews they will be asked to sign a consent form indicating their willingness to participate. Participants for the survey part of the study will be informed of our intention to seek participation for two sets of measures spaced 6–8 months apart and our intention to do this via telephone where possible. Anonymity will be assured to participants. We will expressly ask participants for their anonymised data to be used for all purposes (in this study, to be stored for use in further studies, for teaching and training) and additionally for their permission to seek access to their NHS case records where they exist for the purposes of completing one measure of case complexity. Participants however retain the right at all times to withdraw from the study and not to participate in future investigations.

In acknowledgement of the time commitment made by participants for the case study part of the project we will offer a one-off nominal payment of £10 (in the form of shopping vouchers) as a ‘thank you’ gift to service user and carer/significant other participants. This is clearly stated on information sheets for the study and given to participants following completion of their input to this phase.

### Public and patient involvement

This project was initially conceived with the wider Mental Health Research Network Cymru Service Delivery and Organisation research development group. This group included service users, voluntary organisations, practitioners and academic researchers. The research team included AM a service user researcher who has been involved in the conception and design of the study and will be involved in data collection, analysis, report writing and dissemination.

To oversee the study a project advisory group will meet regularly and include service user members, social care workers, academic researchers and representatives of our lived experience advisory panel. The project advisory group will be independently chaired. The role of this group will be to provide advice to the project team on practical aspects of the study and to consider and provide feedback on study outcomes as they become available.

A lived experience advisory panel (chaired by co-applicant AM) will meet throughout the project and contribute expertise by experience to the process and outcome of the study. Members will be recruited via Involving People (a centrally funded network for supporting public and patient involvement in Wales) to facilitate and support service user involvement on this group.

## Method of analysis

### Quantitative measures

Quantitative data from the standardised measures will be entered into the Statistical Package for Social Scientists software for analysis using parametric and non-parametric techniques (as appropriate, dependent on the nature and distribution of the data, for example) to estimate and explain changes in primary and secondary outcomes over time. Cross-sectional analysis of baseline and follow-up data will be undertaken using wide-format data (in which data for each cases from each time point are entered as a single record) to explain the relationship between Quality of Life (QoL) and intervening variables for service user involvement in decision making, services received and network ties. Changes in QoL of life across time-points will be examined using paired *t*-tests, and explained by linear regression models that include follow-up QoL scores as a dependent variable and baseline QoL scores and other potential covariates such as involvement in decision making as explanatory variables.

### Case study phase

Interviews will be audio-recorded and transcribed in full, with all personal and place identifiers removed. Care plans and other extracts from service user records will be anonymised. Contemporaneous observational field notes will be written up in full, again with all identifiers removed. All items of data will be managed and analysed with the aid of a computer assisted qualitative data analysis software package. Inductive and deductive codes will be created and used to identify and link segments of data in a variety of meaningful ways [36]. We will conduct both within-case (i.e. single trajectory) and across-case analyses [37] to describe in rich detail the unfolding care trajectories of people and the complexities associated with achieving recovery through social care approaches.

## Discussion

This study is located in Wales where devolved control of health and social care has led to changes in mental health policy. Once such change is the introduction of a legal requirement for people in receipt of secondary mental health services to be involved in their care and have recovery focused care and treatment plans. The range of mental health provision however is complex and non-statutory charitable providers also have made significant contributions to the care and support of individuals seeking to recover from mental health problems.

This mixed methods national study of people using non-statutory mental health social services will provide new evidence of how involvement in decisions and building social ties are associated with health and social outcomes for people recovering from mental health problems. The deployment of a range of methods including random national sampling using standardised validated measures and case study interviews of relevant protagonists will give new insights into the everyday experiences of people accessing social care services as they recover from mental ill health.

We will provide new evidence on whether people with mental health problems are in a position to make decisions about their care and treatment. Involvement in decision-making about one’s care appears to be an important contributing factor to achieving recovery and its absence may limit opportunities for self-efficacy. The presence or absence of social support networks may provide indicators of opportunities to establish social connectedness as a key element in recovery [38]. This study will contribute to a growing evidence base on how recovery from mental health problems is planned, provided and co-ordinated [39] and how this relates to quality of life for individuals. We expect to be able to contribute new understandings of the association between involvement in care decisions, social ties and patient reported recovery and quality of life outcomes for people with mental health problems.

## Conclusion

It is clear that biomedical interventions are only one part of a complex web of approaches to help people recover from mental ill health and improve their quality of life. Social approaches have been recognised as crucial to the achievement of better health and social care outcomes. Social approaches themselves constitute a vast range of possibilities in terms of potential avenues to explore. In choosing to focus on involvement in decisions and social ties and their relationship to recovery and quality of life outcomes we have been greatly assisted by previous synthesis of the existing evidence highlighting these as particular areas for fruitful further investigation [38]. This study proposal therefore builds on existing evidence to provide an examination of the relevance and extent of key social approaches to recovery outcomes in mental health. This study offers the possibility of providing new evidence on social responses to mental ill health as a basis for future well designed controlled studies that will enable comparison between Wales and other countries.

## Abbreviations

LSNS: Lubben Social Network Scale
MANSA: Manchester Short Assessment of Quality of Life Scale
MARC2: Matching Resources to Care
NISCHR: National Institute for Social Care and Health Research
QoL: Quality of Life
QPR: Process of Recovery Questionnaire
SDM: Shared Decision Making

## References

[1] Friedli L, Parsonage M (2009). Promoting mental health and preventing mental illness: the economic case for investment in Wales.

[2] Welsh Government (2012). Code of Practice for Parts 2 and 3 of the Mental Health (Wales) Measure 2010.

[3] Anthony WA (1993). Recovery from mental illness: the guiding vision of the mental health service system in the 1990’s. Psychiatr Rehabil J.

[4] Charles C, Gafni A, Whelan T (1997). Shared decision-making in the medical encounter: What does it mean? (or it takes at least two to tango). Soc Sci Med.

[5] Adams J, Drake R (2006). Shared decision-making and evidence-based practice. Community Ment Health J.

[6] Thoits PA (2011). Mechanisms Linking Social Ties and Support to Physical and Mental Health. J Health Soc Behav.

[7] Portes A (1998). Social capital: its origins and applications in modern sociology. Annu Rev Sociol.

[8] Thoits PA (1982). Conceptual, methodological and theoretical problems in studying social support as a buffer against life stress. J Health Soc Behav.

[9] Goldberg RW, Rollins AL, Lehman AF (2003). Social network correlates among people with psychiatric disabilities. Psychiatr Rehabil J.

[10] Lubben J, Gironda M, Phillipson C, Allan G, Morgan DHJ (2004). Measuring social networks and assessing their benefits. Social networks and social exclusion: sociological and policy perspectives.

[11] Webber M, Reidy H, Ansari D, Stevens M, Morris D (2015). Enhancing social networks: a qualitative study of health and social care practice in UK mental health services. Health Soc Care Community.

[12] Sullivan M (2003). The new subjective medicine: taking the patient’s point of view on health care and health. Soc Sci Med.

[13] Charles C, Gafni A, Whelan T (1999). Decision-making in the physician–patient encounter: revisiting the shared treatment decision-making model. Soc Sci Med.

[14] Stacey D, Bennett CL, Barry MJ, Col NF, Eden KB, Holmes-Rovner M, Llewellyn-Thomas H, Lyddiatt A, Légaré F, Thomson R. Decision aids for people facing health treatment or screening decisions. Cochrane Database Syst Rev. 2011;(10):CD001431. doi:10.1002/14651858.CD001431.pub3. 10.1002/14651858.CD001431.pub3 21975733

[15] Elwyn G, Frosch D, Thomson R, Joseph-Williams N, Lloyd A, Kinnersley P, Cording E, Tomson D, Dodd C, Rollnick S (2012). Shared Decision Making: A Model for Clinical Practice. J Gen Intern Med.

[16] Whitaker R (2004). The case against antipsychotic drugs: a 50-year record of doing more harm than good. Med Hypotheses.

[17] Deegan PE (2007). The lived experience of using psychiatric medication in the recovery process and a shared decision-making program to support it. Psychiatr Rehabil J.

[18] Gilburt H, Slade M, Bird V, Oduola S, Craig TK (2013). Promoting recovery-oriented practice in mental health services: a quasi-experimental mixed-methods study. BMC Psychiatry.

[19] Aston V, Coffey M (2012). Recovery: what mental health nurses and service users say about the concept of recovery. J Psychiatr Ment Health Nurs.

[20] Care Quality Commission (2011). Community mental health services survey.

[21] Care Quality Commission (2013). Mental health act annual report 2011/12.

[22] Elias E, Singer L (2009). Review of the care programme approach in Wales.

[23] Wales Audit Office (2011). Adult mental health services: follow up report.

[24] Cohen J (1988). Statistical Power Analysis for the Behavioral Sciences.

[25] Neil ST, Kilbride M, Pitt L, Nothard S, Welford M, Sellwood W, Morrison AP (2009). The questionnaire about the process of recovery (QPR): A measurement tool developed in collaboration with service users. Psychosis.

[26] Corrigan PW, Giffort D, Rashid F, Leary M, Okeke I (1999). Recovery as a psychological construct. Community Ment Health J.

[27] O'Connor AM (1995). Validation of a Decisional Conflict Scale. Med Decis Making.

[28] National Health Service (2012). Measuring Shared Decision Making.

[29] Priebe S, Huxley P, Knight S, Evans S (1999). Application and Results of the Manchester Short Assessment of Quality of Life (Mansa). Int J Soc Psychiatry.

[30] Huxley P, Reilly S, Gater R, Robinshaw E, Harrison J, Mohamad H, Butler T, Windle B (2000). Matching resources to care: the acceptability, validity and inter-rater reliability of a new instrument to assess severe mental illness (MARC-1). Soc Psychiatry Psychiatr Epidemiol.

[31] Huxley P, Reilly S, Robinshaw E, Mohamad H, Harrison J, Windle B, Butler T (2003). Interventions and outcomes of health and social care service provision for people with severe mental illness in England. Soc Psychiatry Psychiatr Epidemiol.

[32] Strauss A, Fagerhaugh S, Suczek B, Wiener C (1985). Social organization of medical work.

[33] Hannigan B, Allen D (2011). Complex Caring Trajectories in Community Mental Health: Contingencies, Divisions of Labor and Care Coordination. Community Ment Health J.

[34] Lipsky M (1980). Street-level bureaucracy: Dilemmas of the individual in public service.

[35] Coffey M (2011). Resistance and challenge: competing accounts in aftercare monitoring. Sociol Health Illn.

[36] Coffey A, Atkinson P (1996). Making sense of qualitative data: complementary research strategies.

[37] Ayres L, Kavanaugh K, Knafl KA (2003). Within-case and across-case approaches to qualitative data analysis. Qual Health Res.

[38] Tew J, Ramon S, Slade M, Bird V, Melton J, Le Boutillier C (2012). Social Factors and Recovery from Mental Health Difficulties: A Review of the Evidence. Br J Soc Work.

[39] Simpson A, Hannigan B, Coffey M, Barlow S, Cohen R, Jones A, Všetečková J, Faulkner A, Thornton A, Cartwright M (2016). Recovery-focused care planning and coordination in England and Wales: a cross-national mixed methods comparative case study. BMC Psychiatry.

